# Enhancing radiographic interpretation: effects of gamification on medical students' knowledge, skills, and satisfaction — a quasi-experimental study

**DOI:** 10.1186/s12909-025-07523-x

**Published:** 2025-07-01

**Authors:** Zahra Karimian, Mohammad Momeni, Nahid Zarifsanaiey

**Affiliations:** 1 Department of E-Learning in Medical Sciences, Virtual School and Center of Excellence in e-Learning, Shiraz University of Medical Sciences, Shiraz, Iran; 2 Division of Radiology, Sina Hospital, Tehran University of Medical Sciences, Tehran, Iran

**Keywords:** E-learning, Online learning, Gamification, Radiology, Radiographic interpretation, Medical education, Clinical Education

## Abstract

**Background:**

Interpreting radiographs is a complex task due to the diverse pathologies that can affect their appearance, leading to difficulties and ambiguities in interpretation. It is crucial to employ engaging and effective educational methods for medical students. This study focuses on designing, implementing, and evaluating a gamification approach to enhance radiographic interpretation skills among medical students.

**Methods:**

The research utilized a Quazi-experimental design involving 82 fifth-year medical students enrolled in a radiographic interpretation course during the 2022–2023 years. Participants were divided into two groups of received internship in a routine method (In-person rotation) and gamified approach training (In-person rotation + Gamification). Students engaged in a competitive gamified environment with a total of 200 clinical cases. Three assessment instruments were used: 80 multiple-choice questions (MCQs), 10 scenario-based cases in an Objective Structured Clinical Examination (OSCE), and a 21-item Technology Acceptance Model (TAM) questionnaire to evaluate student satisfaction. Data analysis was conducted using paired t-tests, independent t-tests, and one-sample t-tests with SPSS version 24.

**Results:**

The comparison of knowledge scores between post-tests and pre-tests indicated significant differences for both routine method (In-person rotation) (*P* < 0.001) and the gamification approach (*P* < 0.001). However, when comparing post-test scores between the two groups, the results revealed that the gamification method was significantly more effective than In-person rotation (*P* < 0.001). Additionally, the acceptance of technology, as measured by student satisfaction in the gamified education group, exceeded the cutoff average across all components.

**Conclusion:**

The study's findings demonstrate that gamified method effectively enhances knowledge, skill, and student satisfaction. Implementing this approach in educational settings can lead to significant benefits, creating a more engaging and interactive learning experience that improves academic outcomes and increases student motivation and enjoyment.

## Introduction

Clinical skill assessment is a crucial part of medical education, directly linked to teaching methods and their improvement. Enhancing practical teaching approaches is essential for raising the quality of medical education [1]. One of the educational courses for medical students is the interpretation of radiographic images, with medical students being a primary target group for radiographic interpretation training. Early diagnosis and treatment are crucial for patient recovery, as they enable life-saving interventions in emergencies [2]. Patients whose radiographic results are interpreted promptly tend to have better treatment processes and recovery periods. However, radiographic interpretations may contain errors, especially during busy workloads or when knowledge is lacking [3].

Radiographic interpretation is a challenging and skilled task due to the vast diversity in patient anatomy and the wide range of pathologies, which can present differently on a single radiographic image. This variability creates ambiguity and difficulty in interpretation [4, 5]. Although imaging technology has improved considerably over the past decades, the frequency of interpretive mistakes has remained constant [6]. Reading and interpreting radiographs is a complex skill that remains essential despite advances in imaging technologies like computed tomography (CT), high-resolution CT, and magnetic resonance imaging (MRI). Accurate interpretation requires knowledge of imaging techniques, anatomy, physiology, and a strong review process. However, it is prone to perceptual and cognitive errors, highlighting the need for comprehensive and ongoing education [5].

Evidence shows that when interpreting radiographs, physicians often express concern about missing lesions. The most significant errors are actually false negative interpretations, although false positives and misclassifications also occur [7]. Decreased confidence in radiographic interpretation stems from the complex mindset required and the many potential sources of error. Understanding these errors is crucial to reducing them and enhancing diagnostic accuracy. Additionally, because radiographs are two-dimensional, black-and-white images, their interpretation is a cognitive and often subjective process, which can also lead to mistakes [8]. Errors in the interpretation and diagnosis of radiographic images can be categorized into several types: technical, perceptual, and analytical (judgment/cognition/decision-making) [8–11]. Each of these errors in interpretation and diagnosis can lead to significant clinical consequences [5]. Therefore, proper training for medical students and residents is crucial in reducing these errors and their resultant implications. Furthermore, advancements in image interpretation in radiography can lead to reduced patient wait times, assurance of patient safety, and cost reductions [12]. Proper training for students in accurate radiographic interpretation is of great importance, as initial diagnoses, especially for general practitioners, can help reduce complications and interpretive errors. Various methods have been used for student education [4, 5, 13, 14].

In recent years, e-learning has gained significant attention, encompassing a wide range of learning strategies and diverse training based on the use of electronic tools and infrastructures [1, 3, 15]. Benefits of e-learning include free access to education, flexibility in time and location for courses, and savings in time and costs [16]. Numerous studies have shown that students are more satisfied with e-learning, which increases motivation for study and effective learning compared to traditional methods such as lectures [17–20]. One innovative educational approach and strategy is gamification, defined as the process of adding game elements to encourage participation and learning [21]. Gamification refers to the use of game elements in non-gaming environments [22, 23]. Gamification enhances students' confidence and motivation by engaging them not only in memorizing information but also through interactive activities [24]. It is increasingly utilized in educational settings due to its potential to make learning enjoyable, memorable, and more effective. Additionally, it offers new and exciting approaches that have yet to be fully explored and utilized in medical education [25].

Theoretical foundations, such as Experiential Learning Theory (ELT) and Self-Determination Theory (SDT), confirm and support the use of gamification in medical education.

Experiential Learning Theory, as developed by Kolb [26], posits that learning is most effective when it is an active, cyclical process involving four stages: concrete experience, reflective observation, abstract conceptualization, and active experimentation [26, 27]. In this model, learners gain knowledge by engaging directly in experiences, reflecting on them, conceptualizing new ideas, and then applying those ideas in practice. This approach is particularly relevant to gamified educational environments, where students participate in interactive activities that mirror real-world challenges, thereby facilitating deeper learning and retention [28].

Self-Determination Theory provides a complementary framework for understanding motivation within gamified learning contexts. SDT emphasizes the importance of fulfilling three basic psychological needs—autonomy, competence, and relatedness—to foster intrinsic motivation and engagement [29, 30]. In gamification, elements such as goal setting, learner control, and feedback can be purposefully designed to support these needs. For instance, allowing students to choose their learning paths (autonomy), providing opportunities to demonstrate mastery (competence), and encouraging collaboration (relatedness) can enhance motivation and learning outcomes [30, 31]. When gamification aligns with these principles, it moves learners toward greater intrinsic motivation and sustained engagement [31].

Also, many studies have confirmed that gamification enhances educational environments through elements such as goals, rules, entertainment, excitement, feedback, rewards, progress, and narrative [32–34]. Although dozens of gamification elements are recognized as effective in creating engagement in classrooms, experts in the field of educational gamification agree that three elements—points, badges, and leaderboards—are common and essential in gamification design [22]. Gamified education is also a very effective method for behavioral and performance changes in students [24, 35, 36].

Medical education serves as an appropriate setting for implementing gamification, particularly with the rise of mobile technologies that have the potential to enhance student learning experiences in educational environments. These technologies can be utilized to improve educational outcomes and elevate the quality of healthcare [37]. One significant area where gamification can greatly improve education is in problem-solving within clinical scenarios. Considering the characteristics of medical sciences and radiology, the integration of gamification could lead to favorable results [38]. The interpretation of radiographs involves substantial problem-solving, and teaching this skill necessitates repeated practice with a variety of cases, where game elements can be advantageous. Various studies have investigated the incorporation of games in radiology education or radiographic interpretation [39–47].

The use of game-based methods in radiology education has historical roots, as demonstrated by Roubidoux et al. (2002), who evaluated a web-based game designed to teach breast imaging to medical students. Through pre- and post-game assessments along with satisfaction surveys, the study revealed that this approach was more engaging, persuasive, and practical, resulting in increased learner satisfaction. As innovative educational strategies in medical sciences progressed, subsequent research in radiology has built upon this groundwork [39].

Ogura (2018) demonstrated that the implementation of a gamified e-learning platform resulted in nearly a twofold increase in the accuracy of third-year medical students when interpreting chest radiographs, particularly for intricate findings like ground-glass opacities. This notable enhancement underscores the effectiveness of gamification in improving diagnostic skills within radiology [40]. Wentzell et al. (2018) evaluated a virtual training model for radiology image interpretation among recent graduates, revealing that the program improved participants' performance, confidence, and interpretive skills. Consequently, over 85% of participants recommended the model for peer use, highlighting its effectiveness in radiology education [41].

In addition to research emphasizing the role of games in radiology education, other studies have specifically explored the effects of gamification in this discipline. Winkel et al. (2020) examined a gamified e-learning platform for radiology training and found it significantly enhanced medical students' diagnostic confidence while lowering error rates [42]. This finding supports the increasing acknowledgment that incorporating game elements into radiology education can improve learning outcomes and boost student engagement. Furthermore, a gamified radiology training module, developed by Staziaki et al. [43] during a 50-h international hackathon, engaged participants in annotating tuberculosis chest radiographs, awarding points based on accuracy, and displaying rankings on a live leaderboard. This approach demonstrated the effectiveness of gamification in enhancing radiology education and won first place among eight competing teams [43].

SonoGames is another innovative gamified competition aimed at improving radiology and point-of-care ultrasound training for emergency medicine residents. Combining simulation and interactive sessions, it addresses delays in adopting modern teaching methods. Survey results indicated that 94% of participants found the competitive format educationally effective, with significant improvements in ultrasound knowledge, enthusiasm, and clinical application reported by residents [44].

While several studies have demonstrated the beneficial effects of games and gamification in radiology education, other research suggests that these methods do not consistently yield significant enhancements in student learning outcomes [45]. Current literature indicates that the application of gamification in radiology remains underexplored, with a systematic review highlighting its limited use across various formats, including board games, physical games, and video games [46]. A recent systematic review conducted in 2024 analyzed 13 relevant studies from nearly 7,000 articles retrieved, emphasizing that while gamification in this field is still limited, it encompasses a diverse range of formats such as board games, physical games, and video games [47]. However, some of the current literature primarily examines in-person gamified environments and often fails to distinguish clearly between pure gameplay (e.g., simulations) and gamification (e.g., point-based systems). This lack of clarity highlights the necessity for more rigorous studies to define terminology and assess context-specific effectiveness.

In our review of both domestic and international sources, we found limited instances of gamification in radiology education. Exploring innovative approaches like gamification could potentially unlock new opportunities for medical education and validate these methods. However, the implementation of gamification in radiology education may vary significantly and produce different outcomes. This research aims to investigate the effects of incorporating gamification into radiographic interpretation education, focusing on three primary objectives:

- ◦ Compare the effects of in-person methods versus gamification methods on medical students' knowledge in radiographic interpretation.
- ◦ Compare the effects of in-person methods versus gamified methods on medical students' skills in radiographic interpretation.
- ◦ Determine medical students' satisfaction with gamified methods of radiographic interpretation.

## Methods

### Study design

The study employed a quasi-experimental design to evaluate the effectiveness of different instructional methods on two groups of fifth-year medical students participating in internships focused on radiographic interpretation at a major medical university in Iran. The first group received routine method (In-person rotation) training through regular attendance in the department, while the second group combined In-person rotation with a gamified approach using the electronic platform Quiz-maker. This research was conducted during the 2022–2023 academic year.

### Participants

The target population for this study consists of fifth-year medical students who have completed a radiology internship course, totaling 82 students. These students are divided into two equal groups of 41 each. A census approach was employed to ensure that all eligible students were included in the research.

### Sample size and sampling

Annually, approximately 160 medical students participate in the radiology interpretation clinical rotation each semester, divided into four groups of around 40 students. Each group undergoes a mandatory two-month rotation in the radiology department under faculty supervision consecutive months. For our study, two of these four groups were randomly selected. The division of medical students in two groups was conducted by the head of the radiology department. In this research each group consisted of 41 students, all matched in age and year of entry. Given that data should not be distributed between the control and intervention groups, random selection was not feasible. Thus, the first group initially completed the routine training method (in-person). Following the conclusion of this training period, the intervention group (routine training combined with gamification) began their rotation.

#### In-person group

Among 82 medical students, the first group (41 students) received in-person internship training by attending the radiology department through rotations in various sections during October–November 2022 and was designated as the control group.

#### Gamification group

The second group (41 students) participated in in-person rotation training combined with gamification during December-January 2022.

#### Inclusion/exclusion criteria

**Fig. 1:**
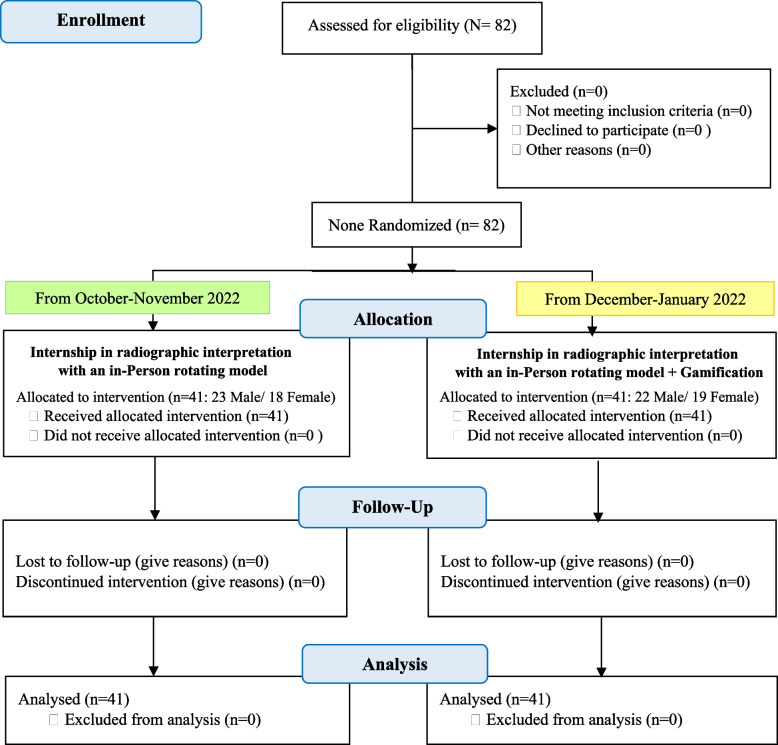
CONSORT flowchart and participant assignment to intervention and control groups

The study involved fifth-year medical students from a medical university who were completing their internship in radiographic interpretation within the radiology department. For the intervention group, it was mandatory for students to have access to a smartphone or laptop/computer to utilize the Quiz-maker platform. Students were excluded from the study if they expressed a lack of willingness to continue participating in the gamification group or if they failed to respond to more than 20% of the questions. A general overview of the grouping and assignment of students into the two intervention and routine groups is depicted in Fig. 1.

### Educational content

Initially, educational content was created across ten topics, each accompanied by 20 questions and clinical cases. The questions featured various formats, including multiple-choice questions (MCQs), matching questions, and problem-based learning (PBL) questions. For the gamification component, real case images were employed, presenting patient images and information in a step-by-step, phased manner within the gamification.

The ten topics covered are as follows:

1. Principles and Techniques of Radiology
2. Chest Radiography
3. Abdominal and Pelvic Radiography
4. Cardiovascular Radiography
5. Identification of Abnormal Calcifications and Their Causes
6. Recognition of Gastrointestinal, Liver, Biliary, and Urinary Tract Abnormalities
7. Bone Radiography
8. Pediatric Radiography
9. Genitourinary Radiography
10. Breast Imaging

The content and outlines were based on the reference textbook *"Learning radiology: recognizing the basics."* [48]. This resource provided foundational knowledge for developing the educational material, ensuring that it aligns with established radiological principles and practices.

### Educational intervention

#### Internship in the in-person rotating group

In the conventional internship program, general medical students engage in theoretical classes and observe as well as analyze radiology cases alongside radiology specialists and clinical assistants for practical learning. The interpretation of radiology films and images is conducted in groups, guided by attending physicians and senior assistants. Theoretical classes take place every morning, following the main reference syllabus. After each class, students are divided into small groups and rotate through different instructors to review educational cases. Initially, students observe the images of each case, note their findings, formulate differential diagnoses, and subsequently present their final diagnoses. They then verify their answers with the instructor, who corrects any mistakes and provides necessary insights.

#### Internship training in the gamification group

In the second group, in addition to the routine methods, educational content was uploaded to the Quiz-maker platform using images, text, and tables based on case studies. Students entered a competitive gamified environment and followed a predetermined learning path, with a total of 200 cases uploaded to this platform.

The gaming environment was designed on the website Quiz-maker, which allows for various question designs, leaderboards, feedback mechanisms, scoring systems, and certificate options. Students were ranked based on their scores, enabling each student to see how they compared to their peers. Students could earn points by answering questions correctly, thereby increasing their scores. Additionally, by completing questions related to each chapter, they could advance their levels and earn extra incentive packages. Each individual had a separate profile where they could track their progress over time.

To further encourage participation, badges were awarded for top skills, and students could receive certificates and rewards. Feedback on questions was provided through reasoned responses and guidance; correct answers were highlighted in green, while incorrect answers turned red. If a student successfully answered at least 70% of the questions correctly, they would automatically receive a certificate at the end of the activity (Medal).

#### Platform

The selection of the Quiz-maker platform was driven by its user-friendly online environment, which facilitates the creation of gamified educational experiences. The training program was organized in a sequential format, featuring questions that consisted of knowledge-based inquiries and clinical scenarios. Each question was accompanied by radiographic images or written explanations, with cases progressing from simple to more complex levels. Students earned points for each correct response, and feedback was provided accordingly. They received a link to access the platform, where they could log in using their names and email addresses. The system allowed for real-time tracking of the number of participants answering questions and their scores, enabling students to gauge their performance against their peers. This competitive element was a significant aspect of the platform, as it generated a leaderboard that encouraged comparison and competition among students.

**Fig. 2:**
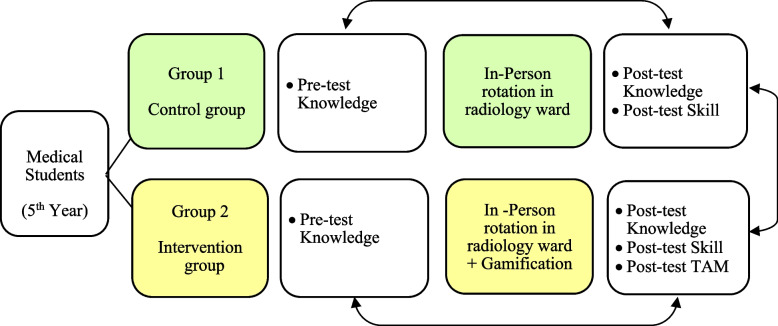
Schematic representation of research stages

Instructors granted virtual badges—gold, silver, and bronze medals—to students who excelled, along with certificates available on the platform. Students logged in with their full names and student IDs, which facilitated a personalized learning experience. This certificate indicated the successful completion of the course. An overview of the intervention methodology for both groups can be seen in Fig. 2.

### Tools/Instrument

In this study, three assessment tools were used to measure knowledge, skills, and satisfaction. Additionally, demographic variables such as gender and age were also collected.

#### Knowledge assessment

At the outset, students were given a pre-test before the course commenced. After the educational intervention utilizing gamification (After two months), a post-test was administered at the course's conclusion. This assessment consisted of 80 multiple-choice questions (MCQs), with a maximum score of 20 points. The passing score was 14, with the minimum moderate score required to pass set at 14 out of 20. The moderate level ranged from scores greater than 14 but less than 17, while achieving a score of 85% (17 or higher) was considered indicative of mastery.

#### Skills assessment

During the course, participants were given clinical cases along with radiographic images. In the routine training group, feedback was provided by attending physicians and senior assistants throughout the course during discussion sessions. Conversely, the gamification group received additional gamified clinical cases presented on the Quiz-maker platform. At the end of the course, students were assessed on 10 clinical cases using the Objective Structured Clinical Examination (OSCE) format. Each case was allotted 6 min, totaling one hour for each student (see Table 1). After answering the questions (cases + radiographic images), the attending physicians explained the answers to the students. Each station was scored out of 2 points, leading to a total score of 20 points. The passing score was established at 70%, equivalent to 14 points. The moderate level ranged from scores of 14 to 17, while achieving a score of 85% (17 or higher) was considered indicative of mastery.

**Table 1:** Titles of OSCE stations

Variables	Title and Cases	Time	Score
Station 1	· Chest radiography-lung mass	6 Minutes	2
Station 2	· Pediatric radiology-diaphragmatic herniation	6 Minutes	2
Station 3	· Abdominal radiology-bowel obstruction	6 Minutes	2
Station 4	· Abdominal calcification	6 Minutes	2
Station 5	· Chest radiography-pneumothorax	6 Minutes	2
Station 6	· Breast mammography-malignancy	6 Minutes	2
Station 7	· Gastrointestinal imaging- achalasia	6 Minutes	2
Station 8	· Bone radiography-bone mass	6 Minutes	2
Station 9	· Genitourinary imaging- tumoral lesion	6 Minutes	2
Station 10	· Cardiac radiography- cardiomegaly	6 Minutes	2
Total	10 Station	60 Minutes	20

#### Satisfaction assessment

To assess satisfaction, we employed the standard Technology Acceptance Model (TAM) questionnaire focused on gamification, which was previously utilized by researchers Zhou and Li (2022) and Al-Adwan et al. (2023) [49, 50].

This questionnaire comprises 21 questions rated on a 5-point Likert scale across seven domains: ease of use, usefulness, enjoyment, focus, control, attitude towards application, and behavior change, with three questions dedicated to each domain. A minimum score of 3 out of 5 is required for the game to be considered suitable. The questionnaire items were tailored to fit the gamified context of radiographic interpretation. The validity and reliability of this questionnaire have been established in studies by Zhou and Li (2022) [49]. In domestic research, the tool's reliability was reported at > 0.872, determined using Cronbach's alpha method with a sample size of 40 and encompassing 21 questions.

### Ethical considerations

All participants provided informed consent to participate in the study. All information was collected anonymously, and confidentiality was maintained during data collection, analysis, and publication of results. The gamified educational content was made available to students in the routine group after the completion of the research.

### Data collection

To evaluate knowledge, a multiple-choice question (MCQ) test comprising 80 questions was utilized, with each question valued at 0.25 points. The tests were conducted in-person, both as a pre-test and a post-test at the course's conclusion, with scores ranging from 0 to 20. For skill assessment, an OSCE featuring 10 stations was employed, where each station was worth 2 points, yielding a total score of 20. This assessment was also conducted in-person at the end of the course. Additionally, to measure satisfaction, a Technology Acceptance Model (TAM) questionnaire was distributed exclusively to the gamification group after the course ended.

### Data analysis

To analyze the differences in scores, paired t-tests were conducted to compare pre-test and post-test results within each group. Independent t-tests were utilized for comparing pre-test scores between the two groups and post-test scores between the two groups. Additionally, a one-sample t-test was employed to assess the status of skill and satisfaction scores.

## Results

### Demographic information

A total of 82 students participated in this study, responding to all sections related to knowledge assessment, skill, and satisfaction. Among the participants, 40 were male and 42 were female. The age range of the students was between 22 and 27 years (Table 2).

**Table 2:** Demographic characteristics of participants

Variable		Gamification	Routine	Statistics	*P*-value
Gender (N)	Male	22	18	χ_2_-0.781	0.377
	Female	19	23		
Age (Mean ± SD)	Min	22	22	t=0.367	0.714
	Max	27	26		
	Mean ± SD	22.90 ± 1.26	23.00 ± 1.14		

The results of the comparison between the two groups indicated that there was no significant difference in gender distribution (*P* = 0.377) and age (*P* = 0.714), suggesting that both groups were homogeneous.

### Analytical findings

#### Comparison of knowledge

Based on the findings from the paired t-test, no significant difference was observed in the pre-test scores between the two educational groups (*P* = 0.71), indicating that the status of both groups was similar before the educational intervention, and the results of the post-test can be attributed to the effect of the intervention. The comparison of post-test and pre-test scores in the routine training group (*P* < 0.001) and in the gamification group (*P* < 0.001) showed a significant difference in post-test scores compared to pre-test scores. However, comparing the post-test scores between the two groups revealed that the gamification method was significantly more effective than the routine method (*P* < 0.001). (Table 3).

**Table 3:** Comparison of pre-test and post-test total scores of knowledges

Variable	Groups	df	t	Average		*P*-value
				Pre-test	Post-test	
Knowledge	In-Person + Gamification	40	38.14	13.08 ± 0.94	18.52 ± 1.14	<0.001
	In-Person	40	19.31	12.56 ± 1.51	16.67 ± 1.45	<0.001
	Between-group comparison	80	--	*P*=0.71, t=1.83	*P*<0.001	--
The score range: 0 to 20	Passing score=14	Moderate level: 14 < x < 17		*Mastery score: 17 < x < 20*		

### Comparison of skills

Based on independent t-test results, the overall skill scores of students in both groups exceeded the expected average of 14. Notably, the gamification training group achieved significantly higher scores than the routine training group (In-person rotation) (*P* < 0.001) (see Table 4).

**Table 4:** Comparison of skill scores in post-test of routine and gamification groups

Variables	N	t	Post-test mean score				*P*-value
			In-Person + Gamification			In-Person	
OSCE all mean score	82	7.62	18.51 ± 0.74			16.47 ± 0.74	<0.001
The score range: 0 to 20	Passing score=14		Moderate level: 14 < x < 17			Mastery score: 17 < x < 20	

According to Table 4, both approaches effectively enhanced students' understanding of radiograph interpretation. Nevertheless, those who combined the routine method with the gamification platform showed a higher level of mastery.

**Fig. 3:**
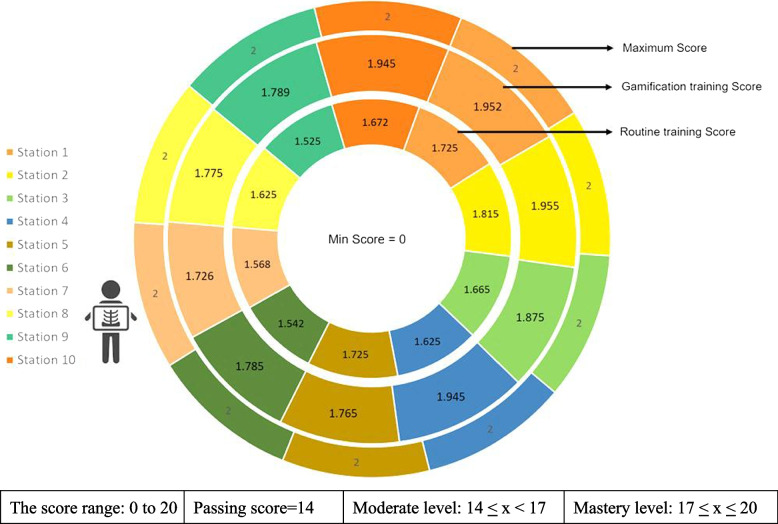
Comparison of skill scores in ten sections of OSCE in routine and gamification groups

Furthermore, the scores of students at each station are depicted in Fig. 3. Each station is evaluated on a scale from 0 to 2, with a total of 10 stations or cases presented, leading to an overall score range of 0 to 20 (see Fig. 3).

The comparison between the two groups at each station reveals that students in the gamification group achieved mastery-level scores across all stations, while the routine training group generally scored below the mastery threshold.

### Students' satisfaction in gamification group

**Fig. 4:**
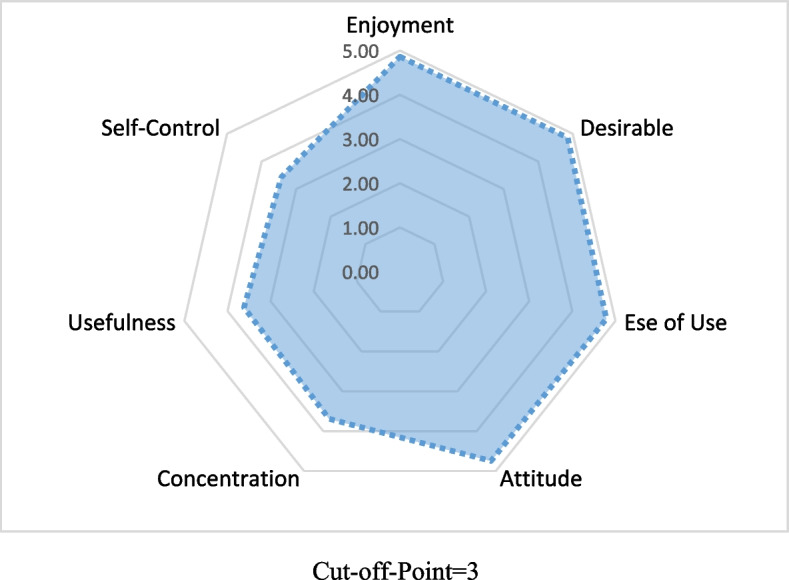
Satisfaction dimensions based on TAM questionnaire in gamification groups

The level of technology acceptance or satisfaction among students in the gamification-based training group was evaluated using the TAM questionnaire, which encompasses seven domains: ease of use, usefulness, enjoyment, focus, control, attitude towards the application, and willingness to engage in future behavior changes. Scores above 3 indicate an acceptable level of satisfaction (Fig. 4).

The results showed that the average scores for all components surpassed 3, with the highest averages found in enjoyment, desirability (willingness to repeat), ease of use, and positive attitude. In general, the gamification approach appears to foster a more engaging and pleasant educational setting for students, potentially enhancing their learning experience.

### Comparison of two groups by contextual variables

Based on the independent samples t-test, the results indicated that there were no significant differences in knowledge scores and skill scores between the research samples when categorized by gender (Table 5). Given that all participants were fifth-year medical students and their age range was not significantly different, there was no necessity to examine the effect of age or year of admission.

**Table 5:** Comparison of knowledge and skill in routine and gamification groups by gender

Variable	Groups		Mean Score		
			G1-In-Person + Gamification		G2-In-Person
Knowledge	Male		18.51 ± 1.11		16.88 ± 1.40
	Female		18.53 ± 1.20		16.50 ± 1.49
	Between-group comparison		t= 0.770, P=0.939		t= 0.848, P=0.401
Skill	Male		18.54 ± 0.730		16.24 ± 1.63
	Female		18.47 ± 0.785		16.66 ± 1.46
	Between-group comparison		t= 0.330, P=0.764		t= 0.879, P=0.385
The score range: 0 to 20	Passing score=14		Moderatelevel: 14 < x < 17		Mastery score: 17 < x < 20

## Discussion

This study evaluated the effectiveness of gamification in enhancing radiographic interpretation skills among medical students, focusing on knowledge acquisition, skill development, and student satisfaction. Our findings indicate that a gamification approach significantly improves both knowledge and skills compared to traditional methods, with high student satisfaction reported. The results align with prior research supporting gamification in medical education [25, 32–34]. The improvement in knowledge scores in the gamification group suggests that elements like points, badges, and leaderboards effectively engage students and enhance knowledge retention. This is consistent with Ogura (2018), who reported a near twofold increase in chest radiograph interpretation accuracy using a gamified e-learning platform [40]. Unlike Ogura's study, which focused on knowledge alone, our research also demonstrates improvements in clinical skills, as measured by OSCE scores.

The enhanced clinical skills in the gamified group suggest that the competitive and interactive environment encourages active knowledge application in clinical scenarios. The OSCE cases required students to integrate knowledge and decision-making, likely refined through practice in the gamified platform. This active knowledge application is essential for developing competent practitioners, supporting the notion that gamification can facilitate this process.

Furthermore, our findings show significant improvements in knowledge and skills, evidenced by differences between pre-test and post-test scores, indicating reduced errors and increased correct responses after gamification. This is consistent with Winkel et al. (2019), which found that gamification in radiology education improved diagnostic confidence and reduced error rates [42]. By comparing our results with Winkel et al., we reinforce the conclusion that gamification effectively boosts confidence and enhances objective performance measures. Unlike previous studies that primarily described gamified platforms, our research provides quantitative evidence of the gamification approach’s effectiveness. For instance, Staziaki et al. (2022) reported positive experiences with the SIIM Virtual Hackathon, a gamified module incorporating points and leaderboards, where collaborative tasks and feedback contributed to a motivating learning environment. While Staziaki et al. emphasized collaboration and feedback in extensive gamified settings [43], our study extends this by demonstrating measurable improvements in both knowledge and clinical skills.

Our findings indicate notable enhancements in knowledge, skills, and satisfaction from gamified training in radiographic interpretation, particularly in light of the educational transformations prompted by the COVID-19 pandemic. Tay et al. (2023) emphasized that the pandemic accelerated the swift integration of digital technologies and alternative teaching methods, including gamification, to tackle challenges in radiography education. Their research suggests that incorporating technologies like virtual reality and gamified platforms can improve clinical skills and optimize learning [51]. Our results lend empirical support to this view, showing that gamification not only boosts engagement but also results in significant advancements in both knowledge and practical skills.

Similarly, Do et al. (2023) found that a gamified online module for medical students led to higher assessment scores than a non-gamified version, without extending completion time. Students reported increased engagement and enjoyment with the gamified module [52]. These results support our findings, highlighting the effectiveness of game elements—like points, badges, and personalized feedback—in enhancing learning and motivation.

Moreover, social media and digital platforms are increasingly significant in radiology education. Koontz et al. (2022) reported that the Twitter hashtag #ASHNRCOTW generated millions of impressions and interactions during the pandemic, with notable follower growth and engagement afterward. Their study concluded that integrating social media with gamification can greatly improve global interaction and communication in radiology education [53]. This underscores the growing importance of digital engagement in medical training, which our gamified platform also addresses.

Moreover, the design and sustainability of gamified programs are crucial for their success. Welbers et al. (2019) found that session limitations effectively prevented overconsumption of gamified content without reducing engagement, and that general feedback was surprisingly more effective than personalized feedback in maintaining player involvement [54]. These insights suggest that careful consideration of gamification features can optimize learner engagement and long-term sustainability, an aspect we considered in our platform design.

Kok et al. (2022) emphasized the importance of combining text, graphics, and interactive elements to reduce cognitive load and maximize learning impact in screen-based digital tools. Their findings support the use of structured gamified programs with engaging presentations to enhance individual learning outcomes [55]. Our study’s positive student satisfaction scores and technology acceptance ratings corroborate the effectiveness of such well-structured gamified environments. Also, previous research by Wentzell et al. (2018) and Roubidoux et al. (2002) demonstrated that gamified and web-based educational models in radiology improve learner confidence, interpretive skills, and satisfaction, with high recommendation rates among participants [39, 41]. Our findings are consistent with these results, further validating the use of gamification in radiographic interpretation education.

The convergence of evidence from our study and recent literature highlights gamification as a powerful educational strategy that enhances knowledge, skills, motivation, and satisfaction in radiology education. Thoughtful design and integration of gamified elements within digital platforms can address contemporary challenges and improve learning outcomes in medical training.

While Our study showed significant enhancements in knowledge, skills, and satisfaction through a hybrid gamified approach. However, it is essential to recognize that not all research has yielded uniformly positive results regarding gamification in medical education. For example, Kirsch & Spreckelsen (2023) conducted a randomized crossover study revealing that a competitive learning program did not significantly improve students' electronic test scores or intrinsic motivation, even though students reported enjoyment and motivation to study. Interestingly, students spent the least time on competitive elements, indicating a preference for individualized or collaborative learning over competition [56]. This contrasts with our findings, where our gamified platform prioritized individual progress and mastery, potentially leading to the increases in knowledge and satisfaction we observed.

Similarly, Lorenzo-Alvarez et al. (2019) compared practical radiology learning in a three-dimensional virtual environment to traditional face-to-face instruction and found no significant differences in knowledge or skill acquisition. However, the virtual setting did enhance collaborative learning and student participation by reducing anxiety and encouraging engagement [57]. This aligns with our high student satisfaction results but differs in knowledge and skill outcomes. Our intervention, which combined lecture-based and gamified elements, created a hybrid model that may have harnessed the strengths of both methods. Additionally, while our platform featured a leaderboard, it aimed to provide individualized feedback rather than promote intense competition, accommodating a broader range of learner preferences.

These studies collectively highlight that the effectiveness of gamification in medical education, particularly in radiographic interpretation, is influenced by various interrelated factors rather than being universally guaranteed. Key factors include the design of gamified elements—whether competitive or collaborative—the level of personalization provided, and how gamification integrates with other instructional methods. Our findings suggest that a hybrid approach, balancing gamified engagement with individualized feedback and traditional instruction, may optimize learning outcomes and student satisfaction in radiology education. Notably, student satisfaction, as measured by the Technology Acceptance Model (TAM) questionnaire, exceeded the average cutoff across all components, indicating a high level of acceptance and positive perception of the gamified method. This finding is crucial, as student engagement and motivation are well-established drivers of effective learning [17–20, 39, 40, 46, 47]. The positive feedback from students reinforces the idea that gamification can create a more enjoyable and engaging learning environment, potentially leading to improved academic performance.

However, it is important to recognize that the impact of gamification is a multivariable and context-dependent phenomenon. Factors such as the mode of delivery (in-person versus electronic), the choice of gaming platform, the specific game elements employed, and even individual learner characteristics-including personality traits and learning preferences-can significantly modulate its effectiveness [46, 47, 58]. This complexity aligns with emerging literature that cautions against one-size-fits-all assumptions regarding gamification's benefits.

Moreover, while gamification can enhance motivation and engagement, it does not inherently guarantee superior learning outcomes unless thoughtfully designed and contextually adapted. For example, overly competitive elements may alienate some learners who prefer collaborative or personalized approaches, underscoring the need for flexible gamification designs. Our study’s hybrid model, which incorporated leaderboards for individual progress without fostering intense peer competition, may have contributed to the positive reception and effectiveness observed.

In sum, while gamification holds considerable promise as an innovative educational strategy, its successful implementation requires careful consideration of design nuances, learner diversity, and pedagogical integration. Future research should continue to explore these variables systematically to refine gamification frameworks that are adaptable, inclusive, and pedagogically sound, thereby maximizing their potential to improve knowledge, skills, and satisfaction in radiology and broader medical education contexts.

## Strengths and limitations of the study

The involvement of experienced educators in the design and implementation of the gamified activities ensures that the content is relevant and aligned with educational objectives. Finally, the positive feedback from participants indicates a strong acceptance of gamification as a valuable educational approach, suggesting its potential for broader application in medical education. Despite the promising findings regarding the effectiveness of gamification in radiology education, this study has several limitations. First, the sample size may not be representative of the broader population of medical students, which could affect the generalizability of the results. Additionally, the study relied on self-reported measures of satisfaction and engagement, which may introduce bias and limit the objectivity of the findings. Furthermore, the duration of the intervention was relatively short, which may not fully capture the long-term effects of gamified training on knowledge retention and skill development. Lastly, variations in the implementation of gamification across different educational settings may lead to inconsistent outcomes, highlighting the need for further research to validate these findings in diverse contexts.

## Conclusion

The findings of this study indicate that gamification is an effective method for enhancing students' knowledge, skills, and satisfaction. Therefore, employing this approach in educational environments can yield significant benefits. The efficacy of this method may stem from gamification elements such as feedback, rewards, peer comparison, and a relative sense of competition among students. However, comparing this research with previous studies shows that the effectiveness of gamification is influenced by various contextual factors. In terms of satisfaction, most studies, including the present one, consistently demonstrate that gamification creates a motivational environment, engaging students in the educational process and fostering enjoyment in learning. The nature of radiology concepts, particularly radiographic interpretation—due to its reliance on images, problem-based questioning, and interpretation (which allows for multiple pathways), along with case diversity and the potential for trial and error—aligns well with the principles of gamification. Thus, integrating these two approaches can be effective in creatively designing radiology concepts. However, given the impact of various variables on the effectiveness of radiology education, further research in this area is necessary.

## Data Availability

Yes, we have research data to declare. The datasets used and/or analyzed during the current study are available from the corresponding author upon reasonable request.

## Abbreviations

TAM: Technology Acceptance Model
OSCE: Objective Structured Clinical Examination
MCQ: Multiple Choice Question
